# Associations between Dairy Herds’ Qualitative Behavior and Aspects of Herd Health, Stockperson and Farm Factors—A Cross-Sectional Exploration

**DOI:** 10.3390/ani12020182

**Published:** 2022-01-13

**Authors:** Asja Ebinghaus, Katharina Matull, Ute Knierim, Silvia Ivemeyer

**Affiliations:** Farm Animal Behaviour and Husbandry Section, University of Kassel, Nordbahnhofstr. 1a, 37213 Witzenhausen, Germany; katharina.matull@web.de (K.M.); uknierim@uni-kassel.de (U.K.); ivemeyer@uni-kassel.de (S.I.)

**Keywords:** dairy cattle, qualitative behavior assessment, affective state, emotional state, animal welfare, cow welfare, human-animal relationship, test day data

## Abstract

**Simple Summary:**

Cows are sentient beings whose welfare depends on their physical health and how they perceive their living conditions. One scientific approach to this emotional realm is the method of qualitative behavior assessment (QBA) which relies on the animals’ body language expressing their affective state. In the present study we were interested whether important health impairments of dairy cows—udder infections and metabolic imbalances—and external aspects including housing, management, and human-animal contact might be associated with the qualitative behavior of the herds. For this purpose, QBA was carried out on 25 dairy farms. Farm data were recorded via interviews and observations, and health data retrieved from official milk recording reports. We found no associations between the included herd health indicators and QBA. However, herds on farms with deep bedded cubicles or straw yards expressed a more positive emotional state compared to farms with raised cubicles. Additionally, on farms with more voluntary stockperson-cow contacts and restraint of cows during main feeding times, possibly reducing competition, more positive QBA scores were found. These results underline that improved housing conditions and more intensive animal care are related to the emotional aspect of animal welfare.

**Abstract:**

The affective state is an integrated aspect of farm animal welfare, which is understood as the animals’ perception of their living environment and of their internal biological functioning. The aim of this cross-sectional study was to explore animal-internal and external factors potentially influencing dairy cows’ affective state. For this purpose, qualitative behavior assessments (QBA) describing the animals’ body language were applied at herd level on 25 dairy farms. By means of principal component analysis (PCA), scores of PC1 (QBAscores) were determined for further analyses. From monthly milk recordings (MR) one year retrospectively, prevalences of udder and metabolic health impairments were calculated. Factors of housing, management, and human-animal contact were recorded via interviews and observations. A multivariable regression was calculated following a univariable preselection of factors. No associations were found between MR indicators and QBAscores. However, more positive QBAscores were associated with bedded cubicles or straw yards compared to raised cubicles, increased voluntary stockperson contact with the cows, and fixation of cows during main feeding times, the latter contributing to the explanatory model, but not being significant. These results underline the importance of lying comfort, positive human-animal relationship and reduction of competition during feeding for the well-being of dairy cows.

## 1. Introduction

Farm animal welfare is of multidimensional nature and constituted by reciprocally interacting aspects of biological functioning and affective state [1,2,3,4]. The animal’s affective state reflects their perception of the internal functional state and external life circumstances (review in [2]). It is expressed, on the one hand, by the extent of negative experiences, such as pain, suffering or persistent distress, and, on the other hand, by the extent of positive sensations [5,6,7].

In dairy farming, important external factors influencing affective components of cow welfare include the accessibility of resources, herd management, and quality of human-animal interactions: With limited space and competition for resources such as food or lying places, increased agonistic interactions play a role in herd social behavior, which in turn can result in reduced resting and feeding times [8,9], and increased prevalence of integument alterations [10,11]. The relationship between animals and humans is crucial due to regular and partly intense interactions in the daily routine of dairy farming. Various studies have shown that positive experiences with stockpersons directly reduce the cows’ level of fear of humans, or that the presence of a familiar person can have stress-reducing effects during potentially negative procedures, such as veterinary treatment or restraint of the animals (reviews in [12,13,14,15]).

Health impairments like mastitis, lameness, and metabolic disorders belong to the most important production diseases of dairy cows [16], are common causes of culling [17,18], and may also affect the animals’ affective state. While information on lameness prevalences requires animal-based on-farm recording, udder health and risks for metabolic disorders can more easily be monitored via regular milk recording [16,19,20].

The question of how to capture and describe affective states in animals has been intensely debated for decades [21]. Approaches range from the application of neural, behavioral, or physiological indicators, from which conclusions about discrete emotions are drawn, to dimensional concepts that seek to integrate different aspects of emotions into evaluations of valence and arousal or that differentiate short-term emotions and long-term moods [22,23,24]. However, the use of a number of different indicators within one study to capture a more holistic picture of the animals affective state has practical limitations under on-farm conditions, and can also pose the risk of false positives and less reliable conclusions [23]. An alternative holistic ethological method for assessing animals’ affective states under on-farm conditions is provided by qualitative behavioral assessment (QBA) [5,25]. This approach was developed substantially by Wemelsfelder et al. [26,27] and first applied to dairy cows in 2006 [28]. QBA claims to enable direct access to the animals’ affective state through a holistic observation of their expressive behavior, i.e., their entire body language. Compared to quantitative methods, QBA allows the behavioral repertoire of an animal or herd to be considered in its whole and in the context of the environment, rather than considering individual aspects of behavior separately [26,27,29,30]. The extent to which this approach meets scientific quality criteria has been extensively investigated, with many studies especially on questions of reliability (e.g., [28,31,32,33,34]). Observer biases, e.g., due to background knowledge, cannot be excluded. With regard to the intra- and inter observer reliability, however, acceptable to good agreements were achieved in most cases (review in [35]).

Since 2009, QBA has been used within the framework of the Welfare Quality^®^ protocol to assess the affective state of cattle at herd level [36]. Individual studies have examined associations of QBA in cattle with potential internal and external influences (dairy cows: [37,38,39,40,41], calves: [42,43]). In a cross-sectional study on 194 Dutch dairy farms, de Vries et al. [37] investigated potential associations between routinely recorded production and health data and various cow herd level welfare indicators including QBA: worse QBA index scores were associated with deviations in fat:protein ratios and lower urea levels in milk. Against expectation, increased SCC (at 120–210 days in milk) were related to enhanced QBA index scores. No associations were found with indicators relating to fertility, mortality, and milk production [37]. In an experiment with induced *E. coli* mastitis in six dairy cows, de Boyer des Roches et al. [38] found associations between indicators of inflammation (somatic cell scores, microbial findings) and QBA: the higher the inflammation scores, the more negative the valence of the animals’ expression. With regard to environmental influences, herds in loose housing compared to tethered housing exhibited a more positive affective state [37,39]. The same was true for herds in tethered housing when provided with access to an outdoor run [41] or herds with increased grazing for several hours compared to zero-grazing [40].

With respect to dairy calves of an age of 0 to 180 d, on farms with higher numbers of calves as well as organic compared to conventional farming, more positive qualitative behavior was recorded at group level [42]. In another study including dairy calves and young stock, Ellingsen et al. [43] explored associations between different stockperson handling styles and the animals’ qualitative behavior. The authors found more positive valences of calf body language on farms with a calm/patient handling style or more positive stockperson-calf interactions compared to farms with dominating/aggressive or nervous handling styles [43]. In dairy cows, the stockperson influence on the qualitative behavior of the herd has not yet been specifically investigated, nor has it been considered in the interplay with other influencing factors.

Thus, the aim of this cross-sectional study was to explore potential associations between animal-based health aspects (udder and metabolic health) as well as external factors (herd, housing, management, human-animal contacts) and dairy herds’ affective state measured by means of QBA.

## 2. Materials and Methods

### 2.1. Farms and Animals

The data used were collected within the framework of two collaborative projects: the European CORE Organic Plus project ‘OrganicDairyHealth’ [44] and a German national project within the interdisciplinary LOEWE research cluster ‘Animals–Humans–Society’ [45,46]. In the present examination, data of 25 organic dairy farms located in Middle and Northern Germany were included. Farm visits were always carried out in the winter period (in 2015 and 2015/16) in order to allow observations of cow behavior in the barn and to be able to exclude direct influences of grazing.

Although the farm sample was a convenient sample, some inclusion criteria were considered: the farms should mainly keep Holstein Friesian or Red Holstein cows (>50% of the animals in the herd) in loose housing systems (cubicles or straw yards) and participate in official milk recording schemes. Apart from these criteria, a typical range of different farm types should be covered, especially with regard to herd sizes and milking systems. Herd sizes ranged from 29 to 161 cows (mean ± standard deviation (sd) = 69.4 ± 30.8). Five farms used automatic milking systems (AMS), the others milked in fishbone (16 farms) or tandem parlors (four farms). The average daily milk yield per cow ranged from 13.6 to 25.9 kg (mean ± sd = 21.7 ± 2.8). Horned cows were kept by 12 farms, the others kept dehorned or partly genetically hornless cows. Summer pasture was offered for all cows on 23 farms, on one farm only for dry cows, and one farm had zero grazing. The majority of farms (16 farms) were family operated, while the others were farm communities.

### 2.2. Data Collection

#### 2.2.1. Qualitative Behavior Assessment

The cow’s affective state was scored by means of qualitative behavior assessment (QBA) at herd level. QBA was carried out using the fixed list of 20 descriptors from the Welfare Quality^®^ protocol for cattle [36]. On all farms, the observations for the QBA were carried out by the same trained person (experienced in behavior observations of dairy cattle). Observations were always conducted in the afternoon; on farms with milking parlor at the latest 30 min before the afternoon milking. To test for inter-observer reliability, on eight farms an additional qualified experimenter conducted the assessments at the same time.

Depending on herd size and barn structure, two to five observation points were firstly selected in order to be able to include all barn areas where the dairy cows were kept. Afterwards, the herd’s expressive quality of behavior was observed from the observation points for a total of 20 min. Scoring of the 20 descriptors was subsequently done using the software QBA App 1.0.7 (www.egenes.co.uk/qba, accessed on 16 March 2016) on Sony Xperia Z2 tablet computers (sourced in Kassel, Germany). The degree of the expression of each descriptor was marked on a visual analogue scale, each defined with a minimum of 0 mm at the left end and a maximum of 125 mm at the right end of the scale.

**Figure 1:**
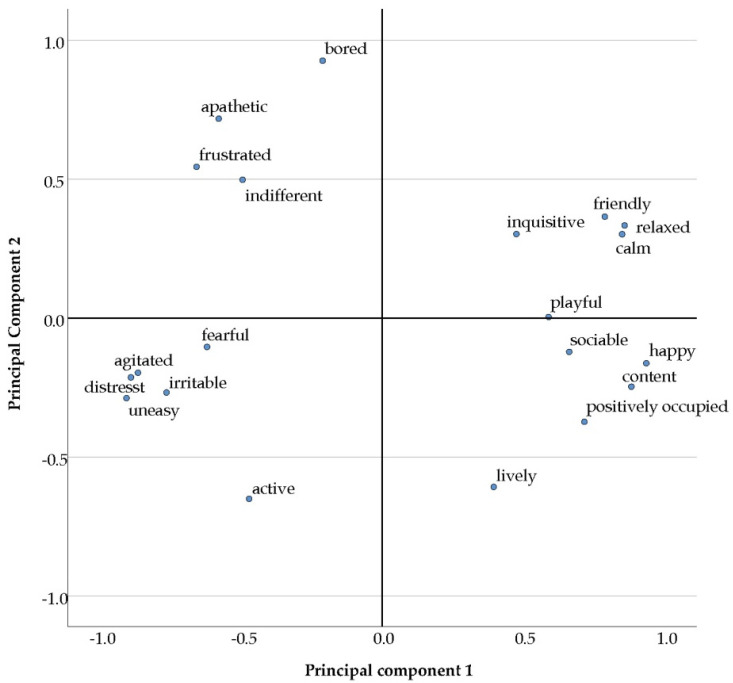
Principal component plot, showing the loadings of the 20 QBA descriptors on the first and second principal component, extracted based on a correlation matrix, without rotation (*n* = 25).

After completion of all farm visits, the multivariate QBA data were reduced to dimensions by a principal component analysis (PCA, based on correlation matrix, without rotation, eigenvalue > 1, SPSS 27). The PCA identified four principal components (PCs), with PC1 explaining the largest proportion of variance between the herds (49.9%), followed by PC2 explaining 18.1%. On PC1, especially those descriptors describing the valence of the behavior loaded strongly (e.g., ‘distressed’, ‘agitated’ in the negative range, and ‘happy’, ‘content’ in the positive range, Figure 1). Descriptors on PC2 tended to express the level of arousal (from e.g., ‘active’ to ‘bored’, Figure 1). PC3 and PC4 contributed only small shares (8.9% and 6.4%, respectively) to the explained variance. In compliance with Wemelsfelder et al. [30] only the factor scores (method: regression, SPSS 27) of PC1 and PC2 were considered further. Inter-observer agreement between two observers was high for the PC1 scores (r_s_ = 0.95, *p* < 0.001, *n* = 8), but there was no agreement regarding PC2 scores (r_s_ = 0.07, *p*= 0.867, *n* = 8). Therefore, only the PC1 scores were used for further statistical analyses.

#### 2.2.2. Cows’ Udder Health and Metabolic Situation

Using data from monthly milk recording data on cow level, herd values were calculated regarding udder health and risks for metabolic disorders.

Udder health was described using a long-term and a short-term indicator calculated from somatic cell counts in milk (SCC): the average percentages of cows with elevated SCC indicating inflammatory processes (SCC ≥ 100,000 [47]) were calculated (1) over one year retrospectively to the farm visit, and (2) for the test day within +/−14 days after/before QBA was recorded.

The fat:protein ratio (FPR) in milk was used as an indicator for cows’ risk of metabolic disorders [48]. At herd level, the proportion of cows in early lactation (≤100 days postpartum) with a risk for ketosis by an energy deficit (FPR > 1.5) and also the proportion of cows with a risk of rumen acidosis (FPR < 1.1) were calculated.

#### 2.2.3. Herd, Housing, Management, and Human-Animal Contact

Herd characteristics, housing and management factors, and human-animal contacts were assessed via questionnaire-guided interviews and observations during the farm visits. With regard to human-animal contacts, the number of cows per stockperson, contact time ‘on foot’ per cow (including milking), active habituation of heifers to humans (e.g., by means of regular udder control before calving, feeding by hand, leading the animals by rope), and the frequency of voluntary contacts beyond routine work were recorded. These voluntary contacts were quantified on the basis of the weekly frequencies of various interactions given as possible answers to the farmers in the interview: observing, brushing, talking to animals, and udder control beyond milking. Each interaction was multiplied by a factor, depending on the frequency stated by the farmers: factor 4 = daily, factor 3 = every 2–3 days, factor 2 = once a week, factor 1 = rarer, factor 0 = never. Thus, each farm could reach up to 16 points. The measure was expressed as the percentage of actual points in relation to the maximal points. In addition to intensity and frequency of contacts, stockpersons’ ability to identify all individual cows was observed during the farm visits.

### 2.3. Statistical Analyses

To identify human and farm factors influencing the herds’ affective state, analyses on herd level were conducted using R 6.3.1 (R Core Team, 2019, R Foundation for Statistical Computing, Vienna, Austria). Because the QBA factor scores of PC1 (QBAscores) were not normally distributed according to the Shapiro-Wilk test (*p* = 0.013), nonparametric procedures were used for the univariable pre-selection of factors: Spearman rank correlations in the case of metric independent variables, and Kruskal-Wallis or Wilcoxon tests in the case of categorical variables. Factors with *p* ≥ 0.1 were selected for subsequent modeling. However, to avoid multicollinearities, these factors were additionally tested for associations among each other using Spearman rank correlations (metric/metric) or effect sizes of Wilcoxon tests (r = $z/\sqrt{n}$, metric/dichotomous), and Cramer’s V of Kruskal-Wallis and Chi^2^ tests (metric/categorical, dichotomous/categorical, categorical/categorical) (Appendix A, Table A1). Strongly related factors (r_s_ > 0.7 or effect sizes r/Cramer’s V > 0.5 [49,50]) were not included together in the model. In those cases, the independent variable with the strongest association with the dependent variable was chosen.

The multivariable linear regression model was fitted using the stepwise selection of factors by Akaike information criterion (AIC) values (model: ‘lm’, function: ‘step, direction = both’). Model diagnostics were done visually by evaluation of the residual distributions and the residuals-by-predicted-values plots. Absence of influential data points was checked using Cook’s distance (≤1.0; [51]). The amount of variance explained through the independent variables was described through the adjusted coefficient of determination (adj. R2). The level of significance used was α = 0.05, and the results are referred to as trends in case of 0.05 < α ≤ 0.10.

## 3. Results

Herd QBA factor scores calculated for PC1 (QBAscores) varied from −2.528 to 1.213 (median = 0.373), with negative values relating to e.g., distress/agitation/irritation, and positive values relating to e.g., relaxation/confidence/positive occupation.

Descriptive data on animal-related health factors, farm characteristics, and the respective results of the univariable analyses are shown in Table 1. Pre-selection resulted in a total of six factors from the areas of herd characteristics, housing, management, and human-animal contacts; none of the health measures showed correlations with QBAscores with *p* ≤ 1.0.

**Figure 2:**
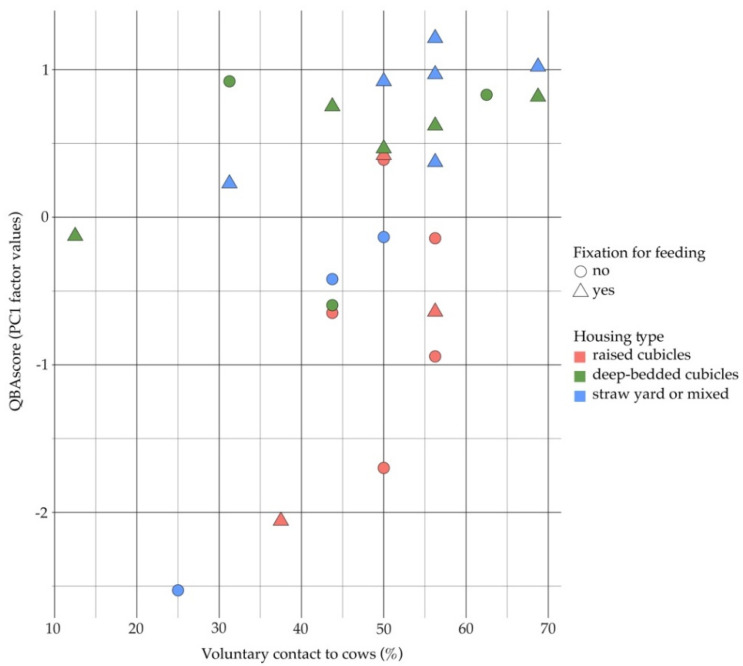
Observed QBAscores (*y*-axis) at different intensities of voluntary contact to cows (*x*-axis) in different housing systems (differentiation in color), with or without fixation for feeding (differentiation in shape), each circle or triangle represents a herd (*n* = 25).

Multivariable modeling yielded a significant final model including three independent factors relating to housing (housing type), human-animal contact (voluntary contact to cows) and feeding management (fixation for feeding) that together explained 42% of variance. The QBAscores (observed values) in the categories/levels of these factors are shown in Figure 2, model estimates and *p*–values are presented in Table 2.

## 4. Discussion

We were interested in exploring potential associations between the affective state of dairy herds expressed in their qualitative behavior (QBAscores) and animal-based health indicators (udder and metabolic health based on somatic cell counts and fat-protein ratios retrieved from milk recording data) as well as farm characteristics regarding herd, housing, management, and human-animal contacts.

### 4.1. Level and Range of the Animal-Based Data

The herds investigated differed markedly, both in terms of their qualitative behavior and in terms of their health state. With regard to QBA, a similar pattern of PC loadings (Figure 1) to those of previous studies using the same set of indicators in dairy herds was revealed [33,34].

With respect to the percentages of cows with elevated SCC, the mean level and range (over one year: median = 50.9, min-max = 35.5–75.8) were comparable to a previous study by Krieger et al. [16] who investigated organic dairy farms in four European countries including Germany (over one year; all: median = 51.3%, min-max = 18.9–94.2%, *n* = 192, DE: median = 53.6%, min-max = 24.8–73.5–75.2%, *n* = 60; values refer to SCC > 100,000). In a recent study (PraeRi) in North, East and South Germany on conventional and organic dairy farms lower percentages of cows with impaired udder health were found based on data from one monthly milk recording report, (medians = 39.3–41.0%, not differentiated between organic and conventional farms, values refer to SCC > 100,000) [17].

Regarding the risks for metabolic disorders, the median percentages of cows within the first 100 days of lactation with a FPR > 1.5, indicating a risk of ketosis, and a FPR < 1.1, indicating a risk for rumen acidosis, were both at a similar level in the investigated herds (FPR > 1.5 = 14.5%; FPR < 1.1 = 12.2%). In comparison, the percentage of cows with a FPR > 1.5 was lower in the study by Krieger et al. [16] (all: median = 10.0%, DE: median = 11.1%), and likewise in the PraeRi study [17] in the three German regions (medians = 8.6–10.7%). Regarding the percentage of cows with FPR < 1.1, comparable data from organic farms are available from a study conducted in Germany and six other European countries [20]: across all countries, the average percentage (mean ± sd = 22.3 ± 1.3, *n* = 128) was higher than in the present study (15.5 ± 9.9); however, on the German farms it was slightly lower at 11.1 ± 7.4 [20].

### 4.2. Associations between Farm Factors and the Herds’ Qualitative Behavior

Multivariable analysis revealed associations between the investigated herds’ QBAscores and quality of the lying area, the quantity of human-animal contacts and, although not statistically significant, fixation of the cows during main feeding times.

Compared to herds on farms with raised cubicles, the qualitative behavior of herds on farms with deep-bedded cubicles, and in tendency also with straw yards (exclusive or additional), was assessed more positively. This effect of housing type may be attributed to the greater comfort of softer and more deformable lying surfaces in deep-bedded cubicles or straw yards compared to raised cubicles, which can only be bedded with smaller amounts of material due to their design. Previous studies have shown that cows prefer lying areas with a softer and well-bedded surface over less or no bedded surfaces (review in [52]). On softer lying surfaces, for instance, standing times were shorter and lying times longer; the number of lying bouts [53,54] and the proportion of cows lying were higher [55]. Besides these effects on cow behavior, the importance of lying surface was also shown in relation to other aspects of animal welfare. In deep-bedded cubicles compared to raised cubicles lower percentages of cows with dirty legs and udders [56], reduced incidences of integument alterations [56,57], and lame cows were found [58]. With regard to straw yards, Fregonesi and Leaver [59] found increased lying and ruminating times, and a greater synchronization of lying behavior compared to cubicle systems. Accordingly, in a preference test, the cows favored straw yards over cubicles, even regardless of space allowance [60]. On the other hand, straw yards compared to cubicle systems present higher risks of dirty udders and impaired udder health [52,54] which was also found on the farms presented here in a related study [44]. Moreover, increased agonistic interactions and subsequent increased restlessness were observed in straw yard systems [61]. In particular the latter is likely to be reflected also in the QBA and may have counteracted positive effects to some degree. In this context, an influence of the milking system is also conceivable: due to the individual animal milking times in the AMS, the lying behavior might be less synchronous [62] and, especially on farms with straw yard, increased restlessness on the lying area might occur. In the present sample, however, a possible interaction between milking and housing system could not be investigated, as no straw yards were used on the participating AMS farms. In future studies, an appropriately balanced selection of farms should be considered.

Concerning the stockperson impact, an association was found with the frequency of voluntary contacts with the cows. These contacts, recorded by interviewing the farmers, included interactions that we assumed the cows perceived as neutral or positive (observing, brushing, talking to animals, and udder control). Accordingly, higher frequencies were associated with higher QBAscores, expressing higher levels of relaxation, confidence, and positive occupation within the herd. Associations pointing in a similar direction were also found in an earlier investigation by Menke et al. [10]: the frequency of brushing was positively associated with the occurrence of affiliative behavior within the herd, which may also contribute to a positive affective state (review in [5]). Possibly, the positive effect of more frequent and intense animal care is mainly attributed to an earlier and better recognition of problems within the herd and to a faster implementation of improvements. In addition, increased human-animal contact of a neutral or positive nature also contributes to less cow fear behavior or physiological stress responses in human-animal interactions (reviewed in [12,13,14,15]), which was confirmed in a related study including the farms presented here within a broader sample [46]. In this and other previous studies, less avoidance behavior of cows towards humans in a standard test were, for instance, related to a closer ratio of cows per stockperson [46,63], increased daily contact time and stockpersons’ ability to identify individual cows [64], and a combined variable including frequency of brushing, identification of cows, gentle handling, number of milkers and frequency of personnel changes [65]. It is conceivable that in dairy farming with its several daily interactions, e.g., around milking, lower fear levels towards humans are also reflected in a generally calmer herd behavior beyond handling situations. Further specific experimental investigations on this would be interesting and relevant for practice.

Lastly, routine fixation of the cows for feeding contributed to model improvement and was included in the final model. The qualitative behavior of herds that were routinely fixed in the feeding gates during main feeding times was assessed more positively, although it was not statistically significant. Routine restraint for feeding after milking can reduce risks of udder infection, especially on farms with straw yard, by preventing the animals from lying on potentially contaminated surfaces with the teat canal still open [66,67]. In fact, in the present study, the mean percentage of cows with elevated somatic cell counts (≥100,000) was numerically lower on farms that routinely restrained cows for feeding (49.8% over one year, *n* = 14) compared to farms without restraint (57.0% over one year, *n* = 11). However, since this was only a statistical trend (Z = −1.807, *p* = 0.071) and no direct associations between QBAscores and udder health indicators was found, this effect is likely less influential. Rather related social aspects may play a role: restraint for feeding has been found to reduce competition for the resource feed and to allow for calmer feeding by avoiding agonistic interactions between animals and resulting injuries, particularly in horned herds [68,69,70]. In the present investigation, a high proportion of horned herds (48%) were involved, and nine out of the in total of 12 horned herds were routinely fixed for feeding. However, in the statistical modeling, the interaction of horn status and fixation for feeding could not be reliably accounted for because of the uneven distribution of factor combinations. Nevertheless, it did provide an indication that the positive effect of fixation for feeding was particularly evident in the horned herds. In order to better describe and quantify the direct influence of social aspects on the cows’ affective state, observations of social behavior should be additionally considered in future studies.

With regard to the herds’ health state, both an influence on the affective state expressed in the qualitative behavior and, conversely, an influence of the affective state on the health situation was expected. However, the health indicators available from the milk recording reports and considered in the present investigation–incidences of FPR deviations and elevated SCC–were not associated with the QBAscores. In order to draw a more comprehensive picture of the influence of biological functioning and health on the animals’ affective state, further disease complexes would have to be included. In addition to udder and metabolic health impairments, claw and limb disorders, for example, are a major health problem in dairy cows [17,18]. Lameness is an expression of pain [71], which is likely to be reflected not only in qualitative aspects of behavior in the affected animals, but also at herd level above a certain prevalence.

Moreover, animal-individual data instead of pooled herd level data might have been more meaningful with regard to the health indicators. While the herd-level QBA is highly feasible in on-farm applications, it does not capture individual differences which may relate to different health states, but also to different coping abilities. Furthermore, the indicators used do not only represent acute clinical diseases, which may be associated with pain or further physical impairments and were related to QBA in a previous study on individual animal level: de Boyer des Roches et al. [38] found that acute mastitis caused by inoculated *E. coli* bacteria had an influence on the QBA scores. However, the proportion of cows with SCC ≥ 100,000 used in the present study also reflects less acute clinical and subclinical mastitis, which is not necessarily painful or limiting to the animals’ general condition.

This also applies to metabolic disorders, which occur more frequently in subclinical than clinical form. Although FPR is an established and widely used measure to identify risks for ketosis and acidosis (e.g., [16,20,37]), the thresholds used and also the method of using milk constituents to identify metabolic disease are debated [72].

In addition to these methodological considerations, limitations of a cross-sectional study must be kept in mind: a range of varying potential influencing factors is set against a limited sample, and therefore only patterns of associations can be detected. Additional experimental investigation under more controlled conditions would be necessary to obtain a picture of causal relationships, and preferably on individual level, to be able to investigate associations between biological functioning and affective state in a more differentiated way.

## 5. Conclusions

From the range of animal, stockperson, and farm factors considered, the importance of cow comfort around resting, increased human-animal contact and reduced competition during feeding was reflected in the affective behavioral expression of the investigated dairy herds. Qualitative behavior scores had a more positive valence on farms with deep bedded cubicles and in tendency straw yards compared to raised cubicles as well as on farms with higher frequencies of voluntary contacts between stockpersons and cows. Additionally, the factor fixation of cows during main feeding times, although non-significant, improved the multivariable model. These results underline that improved husbandry conditions and more intensive animal care are positively related to the affective aspects of animal welfare.

## Figures and Tables

**Table 1:** Pre-selection of factors potentially affecting dairy herds’ behavior described by QBAscores ^1^; based on Spearman rank correlations (r_s_) or group mean differences (Kruskal-Wallis/Mann-Whitney U test); factors selected for multivariable analysis (*p* ≤ 0.1) are marked in blue (*n* = 25).

Animal-Related Factors	Median	Min-Max	r_s_	*p*
% cows with somatic cell count ≥ 100,000, over 1 yr.	50.9	35.5–75.8	−0.29	0.166
% cows with somatic cell count ≥ 100,000, +/− 14 d after/before QBA recording	57.4	34.3–89.7	−0.14	0.512
% cows with fat:protein ratio > 1.5 within the first 100 DIM, over 1 yr.	14.5	3.4–24.6	0.00	0.991
% cows with fat:protein ratio < 1.1 within the first 100 DIM, over 1 yr.	12.2	3.2–43.7	0.08	0.718
**Herd characteristics**	**Median**	**Min-Max**	**r_s_**	***p***
**Herd size**	68	29–161	−0.33	**0.104**
	**Groups**	**Number**	**QBAscore ^1^**	***p***
Horn status	Horned	12	−0.095	0.231
	Hornless ^2^	13	0.103	
**Housing**	**Groups**	**Number**	**QBAscore ^1^**	***p***
**Housing type**	Raised cubicles	8	−0.665	**0.027**
	Deep bedded cubicles	8	0.460	
	Straw yards or mixed ^3^	9	0.182	
Cow:cubicle ratio or lying space (m^2^/cow) ^4^	Suboptimal	10	−0.206	0.273
	Minimum recommendations	11	−0.035	
	Generous	4	0.611	
**Cow:feeding place ratio** ^4^	Suboptimal	7	−0.779	**0.032**
	Minimum recommendations	9	0.276	
	Generous	9	0.330	
**Access to outdoor run**	No (or limited ^5^)	8	−0.430	**0.071**
	Yes	17	0.202	
**Management**	**Median**	**Min-Max**	**r_s_**	***p***
Concentrates (ø kg/cow*year)	1200	0–2000	0.17	0.423
	**Groups**	**Number**	**QBAscore ^1^**	***p***
**Routine fixation of cows for feeding**	No	11	−0.452	**0.025**
	Yes	14	0.355	
Milking system	AMS	5	0.019	0.156
	Fishbone parlor	16	−0.185	
	Tandem parlor	4	0.717	
Separation of dry cows	No	6	0.013	0.703
	Yes	19	−0.004	
Separation of diseased or lame cows	No	7	0.014	0.499
	Sometimes	11	−0.275	
	Yes	7	0.419	
**Human-animal contact**	**Median**	**Min-Max**	**r_s_**	***p***
Number of cows per stockperson ^6^	18.0	4.4–40.5	−0.17	0.413
Contact time per cow ’on foot’ (min/d) ^7^	6.0	1.7–32.6	0.19	0.355
**Voluntary contact to cows (%)** ^8^	50.00	12.50–68.75	0.44	**0.030**
	**Groups**	**Number**	**QBAscore ^1^**	***p***
Active habituation of heifers to humans	No	15	−0.107	0.956
	Yes	10	0.160	
Identification of cows	No	9	−0.036	0.734
	Yes	16	0.020	

^1^ Factor values of the first principal component: positive values relate to relaxation/positive occupation/confidence, negative values relate to distress/agitation/irritation; ^2^ dehorned or genetically polled cows; ^3^ six farms with straw yards, three farms with both straw yard and deep bedded cubicles; ^4^ based on recommendations cited in the literature (details in Appendix A, Table A2 and Table A3); ^5^ one farm only had an outdoor area of about 0.2 m^2^/cows, on another farm only one of two lactating groups had access to the outdoor area; ^6^ incl. herd managers, employees, trainees; ^7^ incl. milking, excl. time near the cows, but on machines; ^8^ percentage of actual points in relation to maximal points allocated to weekly frequencies of different human-animal interactions beyond routine work named by the interviewed farmers, see text (Section 2.2.3).

**Table 2:** Final multivariable linear regression model regarding the outcome variable QBAscore (*n* = 25).

Factors	Estimate ^3^	Standardized ^4^	SE	t ^5^	*p*
(Intercept)	−2.554	0.000	0.659	−3.875	0.001
Housing type (reference: raised cubicles)					
Deep bedded cubicles	1.135	0.540	0.394	2.897	0.009
Straw yard or mixed ^1^	0.751	0.368	0.384	1.958	0.064
Voluntary contact to cows (%) ^2^	0.034	0.446	0.012	2.809	0.011
Routine fixation of cows for feeding	0.493	0.250	0.322	1.534	0.141
adjusted R^2^ = 0.417, F = 5.299, *p* < 0.01, VIF = 1.036–1.100					

^1^ Six farms with straw yards, three farms with both a straw yard and deep bedded cubicles; ^2^ Percentage of actual points in relation to maximal points allocated to weekly frequencies of different human-animal interactions beyond routine work named by the interviewed farmers, see text (2.2.3); ^3^ regression coefficient; ^4^ standardized regression coefficient; ^5^ value of the t-statistic used to calculate *p*–values.

## Data Availability

As this study collected data from commercial farms, some of which are sensitive, none of the data are available in an official repository. On request, however, the data can be made available in anonymized form.

## Appendix A

**Table A1:** Associations between the six factors preselected for multivariable analysis: Spearman rank correlation coefficients (r_s_, metric/metric), effect sizes R of Wilcoxon tests (R, metric/dichotomous) or Cramer’s V of Kruskal-Wallis and Chi^2^ tests (V, metric/categorical, categorical/categorical), ** = *p* < 0.01, * = *p* < 0.05, (*) = *p* < 0.1.

	Housing Type	Cow: Feeding Place Ratio	Access to Outdoor Run	Fixation for Feeding	Voluntary Contact to Cows
Herd size	V = 0.27 (*)	V = 0.20	R = −0.02	R = −0.56 **	r_s_ = 0.44 *
Housing type (raised cubicles, deep bedded cubicles, straw yards)	–	V = 0.38	V = 0.47 (*)	V = 0.26	V = 0.13
Cow:feeding place ratio (suboptimal, minimum recommendations, generous)	V = 0.38	–	V = 0.53 *	V = 0.39	V = 0.19
Access to outdoor run (yes/no)	V = 0.47 (*)	V = 0.53 *	–	V = 0.08	R = −0.28
Fixation for feeding (yes/no)	V = 0.26	V = 0.39	V = 0.08	–	R = −0.18

**Table A2:** Lying space–recommendations for herds with milking parlor (MP) or automatic milking system (AMS) and with horned or hornless cows, and categories for analyses derived from these recommendations.

References/Categories	Cubicles (MP) (Cow: Cubicle Ratio)	Cubicles (AMS) (Cow: Cubicle Ratio)	Straw Yards (Hornless Cows) (m^2^/Cow)	Straw Yards (Horned Cows) (m^2^/Cow)
[74]	1:1		4.5–5	7–9
[75]	1:1		6	8
[76]	1:1	1:0.98 ^1^	8–9	
[77]			≥ 5	8
[68]				8
[78]		1:0.90 ^2^		
Suboptimal	<1:1	<1:0.95	<4.5	<7
Minimum recommendations	1:1–1:1.05	1:0.95–1:1	4.5–6.5	7–9
Generous	>1:1.05	>1:1	>6.5	>9

^1^ own calculation based on [76]; ^2^ own calculation based on [78].

**Table A3:** Feeding places-recommendations for herds with milking parlor (MP) or automatic milking system (AMS) and with horned or hornless cows, and categories for analyses derived from these recommendations.

References	Feed Gates (MP) (Cow: Feeding Place Ratio)	Feed Gates (AMS) (Cow: Feeding Place Ratio)	Feed Rails (Hornless Cows) (Feeding Place Width)		Feed Rails (Horned Cows) (Feeding Place Width)	
[74]	1:1		70–75 cm			
[75,79]			75 cm		85–90 cm	
[76]	1:1	1.5:1	65–75 cm			
[77]			75 cm		85 cm	
[68]	1:1–1:1.1				85–100 cm	
[78]		1.5:1				
[80]	1:1.1				85 cm	
Categories	Feed Gates (MP)	Feed Gates (AMS)	Feed Rails (Hornless, MP)	Feed Rails (Hornless, AMS)	Feed Rails (Horned, MP)	Feed Rails (Horned, AMS)
suboptimal	<1:0.95	<1:0.83	<70 cm	<62 cm	<80 cm	<70 cm
minimum recommendations	1:0.95–1:1.05	1:0.83–1:1	70–75 cm	62–75 cm	80–90 cm	70–90 cm
generous	>1:1.05	>1:1	>75 cm	>75 cm	>90 cm	>90 cm
